# Extreme temperatures increase the risk of pediatric pneumonia: a systematic review and meta-analysis

**DOI:** 10.3389/fped.2024.1329918

**Published:** 2024-02-02

**Authors:** Firdian Makrufardi, Rina Triasih, Nurnaningsih Nurnaningsih, Kian Fan Chung, Sheng-Chieh Lin, Hsiao-Chi Chuang

**Affiliations:** ^1^ International Ph.D. Program in Medicine, College of Medicine, Taipei Medical University, Taipei, Taiwan; ^2^ Department of Child Health, Faculty of Medicine, Public Health, and Nursing, Universitas Gadjah Mada—Dr. Sardjito Hospital, Yogyakarta, Indonesia; ^3^ National Heart and Lung Institute, Imperial College London, London, United Kingdom; ^4^ Department of Pediatrics, School of Medicine, College of Medicine, Taipei Medical University, Taipei, Taiwan; ^5^ Division of Allergy, Asthma, and Immunology, Department of Pediatrics, Shuang Ho Hospital, Taipei Medical University, New Taipei City, Taiwan; ^6^ School of Respiratory Therapy, College of Medicine, Taipei Medical University, Taipei, Taiwan; ^7^ Division of Pulmonary Medicine, Department of Internal Medicine, Shuang Ho Hospital, Taipei Medical University, New Taipei City, Taiwan; ^8^ Cell Physiology and Molecular Image Research Center, Wan Fang Hospital, Taipei Medical University, Taipei, Taiwan; ^9^ Graduate Institute of Medical Sciences, College of Medicine, Taipei Medical University, Taipei, Taiwan

**Keywords:** climate change, meta-analysis, pediatric, pneumonia, respiratory health, temperature

## Abstract

**Introduction:**

The impact of climate change on ambient temperatures threatens to worsen pediatric pneumonia-related outcomes considerably. This study examined the associations of temperature variation and extreme temperature with pediatric pneumonia-related events using a meta-analysis.

**Methods:**

We systematically searched PubMed, Medline, Embase, and Web of Science databases for relevant literature, and the quality of evidence was assessed. Fixed and random-effects meta-analyses were performed to calculate the pooled relative risks (RRs) of the associations with pneumonia-related events.

**Results:**

We observed that a 1°C temperature variation increased the RR of pneumonia events by 1.06-fold (95% confidence interval (CI): 1.03–1.10). A 1°C temperature variation increased the RR by 1.10-fold of the pediatric pneumonia hospital admissions (95% CI: 1.00–1.21) and 1.06-fold of the pediatric pneumonia emergency department visits (95% CI: 1.01-1.10). Extreme cold increased the RR by 1.25-fold of the pediatric pneumonia events (95% CI: 1.07–1.45). A 1°C temperature variation increased the RR of pneumonia events in children by 1.19-fold (95% CI: 1.08–1.32), girls by 1.03-fold (95% CI: 1.02–1.05), and in temperate climate zones by 1.07-fold (95% CI: 1.03–1.11). Moreover, an increase in extreme cold increased the RR of pneumonia events in children by 2.43-fold (95% CI: 1.72–3.43), girls by 1.96-fold (95% CI: 1.29–2.98) and in temperate climate zones by 2.76-fold (95% CI: 1.71–4.47).

**Conclusion:**

Our study demonstrated that pediatric pneumonia events are more prevalent among children, particularly girls, and individuals residing in temperate climate zones. Climate change represents an emergent public health threat, affecting pediatric pneumonia treatment and prevention..

**Systematic Review Registration:**

PROSPERO (CRD42022378610).

## 1 Introduction

The impact of climate change on human health, particularly in the form of increased frequency and intensity of extreme weather, is a growing concern, with children being among the most vulnerable populations affected (1). In 2021, the United Nations Children's Fund (UNICEF) stated that every child globally is subjected to at least one climate-related hazard, shock, or stress, emphasizing the need for heightened action to combat climate change (2). The Intergovernmental Panel on Climate Change (IPCC) has highlighted the substantial threat that climate change poses to respiratory health, including an increased risk of pediatric pneumonia (3). Pneumonia is the leading cause of infectious disease in children and adolescents and is characterized by cough, difficulty breathing, and fever (4, 5). Pediatric pneumonia is most commonly a consequence of *Streptococcus pneumoniae* and *Haemophilus influenzae type b (Hib)* bacterial infection or respiratory syncytial virus viral infection (6). The link between exposure to extreme weather and pneumonia-related outcomes has been investigated in the last few decades (7–9). Temperature variation and extreme temperature, according to accumulating epidemiological evidence, have a significant impact for pediatric pneumonia events and hospitalization (10, 11). This demonstrates that variation and extreme temperature is an important risk factors for pediatric pneumonia.

Temperature variation increase the risk of pediatric pneumonia-related events (12). Several studies have highlighted that temperature variation is an important risk for pneumonia hospital admissions in adolescents (13, 14). In addition, two studies in humid subtropical climate zones have also focused on children as a population at risk of pneumonia hospital admission due to temperature variation (15, 16). Also, studies in tropical climate zones have observed that temperature variation is a critical risk factor in children for pneumonia outpatient visits (17, 18). Several studies in humid subtropical climate zones have also concluded that there are significant associations between 1°C temperature variation and pneumonia outpatient visits in children (19–22). These reports highlights the link between temperature variation and increased risk of pediatric pneumonia events.

Extreme temperatures are also associated with the risk of pneumonia events (23). Extreme heat is a crucial risk factor for pneumonia emergency department visits and pneumonia hospital admissions in children (10, 24). However, extreme heat is also associated with decreased risk of pneumonia events in tropical climate zones (25, 26). In a study in Korea, extreme cold increased the relative risk of pediatric pneumonia emergency department visits by 1.02-fold (9). A study in South America observed that an increase in extreme cold increased the risk of pediatric hospital admissions by 1.12-fold (25). Moreover, extreme cold is an important risk factor for pneumonia outpatient visits among adolescents in tropical regions (27). Taken together, extreme temperatures contribute to diverse and heightened risks of pediatric pneumonia across various geographical regions.

Epidemiological studies have identified evidence of links between extreme weather and pneumonia-related events in children. However, the results appeared inconsistent, and there is still a lack of systematic reviews that have been conducted. The aim of this study is to examine the associations of temperature variation and extreme temperature with pediatric pneumonia-related events using a meta-analysis.

## 2 Materials and methods

### 2.1 Search strategy

The Preferred Reporting Items for Systematic Reviews and Meta-analyses (PRISMA) guidelines were followed to structure this meta-analysis (28). The Meta-analyses of Observational Studies in Epidemiology checklist was also followed (29). This study was registered in PROSPERO before beginning with the registration ID CRD42022378610. We used the following terms: (pneumonia OR respiratory tract infection) and (meteorology OR temperature OR extreme temperature OR extreme heat OR extreme cold OR temperature variation OR ambient temperature OR inter-day temperature variability OR diurnal temperature range OR temperature changes between neighbouring days OR temperature variation between neighbouring days). PubMed, Medline, Embase, and Web of Science were all searched using a Boolean search string (see Supplementary Table S1 in the Supplementary Material). The database searches were most recently updated on March 31, 2023.

### 2.2 Inclusion and exclusion criteria

We included studies meeting the following criteria: (1) the outcome in the study was diagnosis of pneumonia defined according to the *International Classification of Diseases* or local hospital or national records; (2) the study considered various temperature extremes, which encompassed extreme heat (characterized as temperatures at or above the 99th, 90th, or 75th percentiles), extreme cold (characterized as temperatures at or below the 1st, 10th, or 25th percentiles), and temperature variations (intra- or inter-day temperature variability); and (3) the study provided raw data (e.g., frequencies) for computation of odds ratios or relative risks (ORs/RRs) or reported ORs or RRs. Further, we excluded studies meeting the following criteria: (1) reviews, commentaries, or letters; (2) non-English language; or (3) having another focus (i.e., full-text articles without presenting data on review outcomes or temperature exposure); (4) study included cases of pneumonia related to COVID-19.

### 2.3 Study selection

We utilized EndNote software to conduct title and abstract screening as well as the full-text review. Two reviewers independently assessed the relevance of articles based on the titles and abstracts including the temperature search terms. Next, the full-text articles were evaluated based on the inclusion and exclusion criteria. Disagreement were discussed with a third reviewer until a consensus was reached. The corresponding authors were contacted to obtain coefficient estimates, and available data in the manuscript were used when the corresponding authors were unable to provide detailed data.

### 2.4 Data extraction

Information obtained from the selected articles was entered into an Excel spreadsheet. Extracted data consisted of study design, duration of study, sample size, location, age subgroup, and RRs or ORs with 95% confidence intervals (CIs). Study design was classified as cross-sectional, case-control, case-crossover, time series and cohort studies. Location was coded at the country level and included city information. The age subgroups in this study were defined according to the American Academy of Pediatrics, covering children (<13 years) and adolescents (13–18 years) (30). Sex was classified as male or female. The study locations were classified on the basis of the Köppen-Geiger climate classifications of tropical, arid, temperate, cold, or polar (31). Extreme temperature was divided into extreme heat (defined as temperature above the 99th, 90th or 75th percentile etc., in a single day) and extreme cold (defined as temperatures below the 1st, 10th or 25th percentile etc., in a single day) (32). We defined pneumonia events as a pneumonia requiring an unscheduled visit, an ED visit, hospitalization, or outpatient visit. We reported the combined effect of the intra- and interday temperature variations in all seasons on pneumonia risk. We used Joanna Briggs Institute Critical Appraisal Checklist to assess the risk of bias (33). Assessing potential bias through funnel plots was deemed unsuitable in this review due to the limited number of studies incorporated into this meta-analysis (34). Details of assessment of risk of bias are presented in Supplementary Table S2 in the Supplementary Material.

### 2.5 Statistical analysis

In the meta-analysis, we retrieved effect estimate RRs and 95% CIs or computed these from raw data using a practical meta-analysis calculator for associations between temperature and pneumonia. In the meta-analysis, the effect estimates (RRs/ORs and 95% CIs) of the included studies were quantitatively pooled. ORs were considered equivalent to RRs under the rare-disease assumption; we used RR values as measures of associations in the meta-analysis (35). To test our hypothesis for participants who were exposed to extreme temperatures or temperature variations, a random-effects or fixed-effects meta-analysis was used. Heterogeneity between the studies was evaluated using Cochran's Q and *I*^2^ statistics. *p* < 0.10 indicated significance for the Cochran's Q statistic, and *I^2^* values of greater than 50% indicated moderate-to-high heterogeneity (36). If *p* > 0.1 and *I*^2^ > 50%, a fixed-effects model was used; otherwise, a random-effects models was used. To assess the stability of the pooled estimates, a leave-one-out sensitivity analysis was performed. All statistical analysis procedures were performed using Review Manager software (RevMan version 5.4.1). Statistical significance was judged by *p* value of <0.05.

## 3 Results

### 3.1 Study characteristics

**Figure 1:**
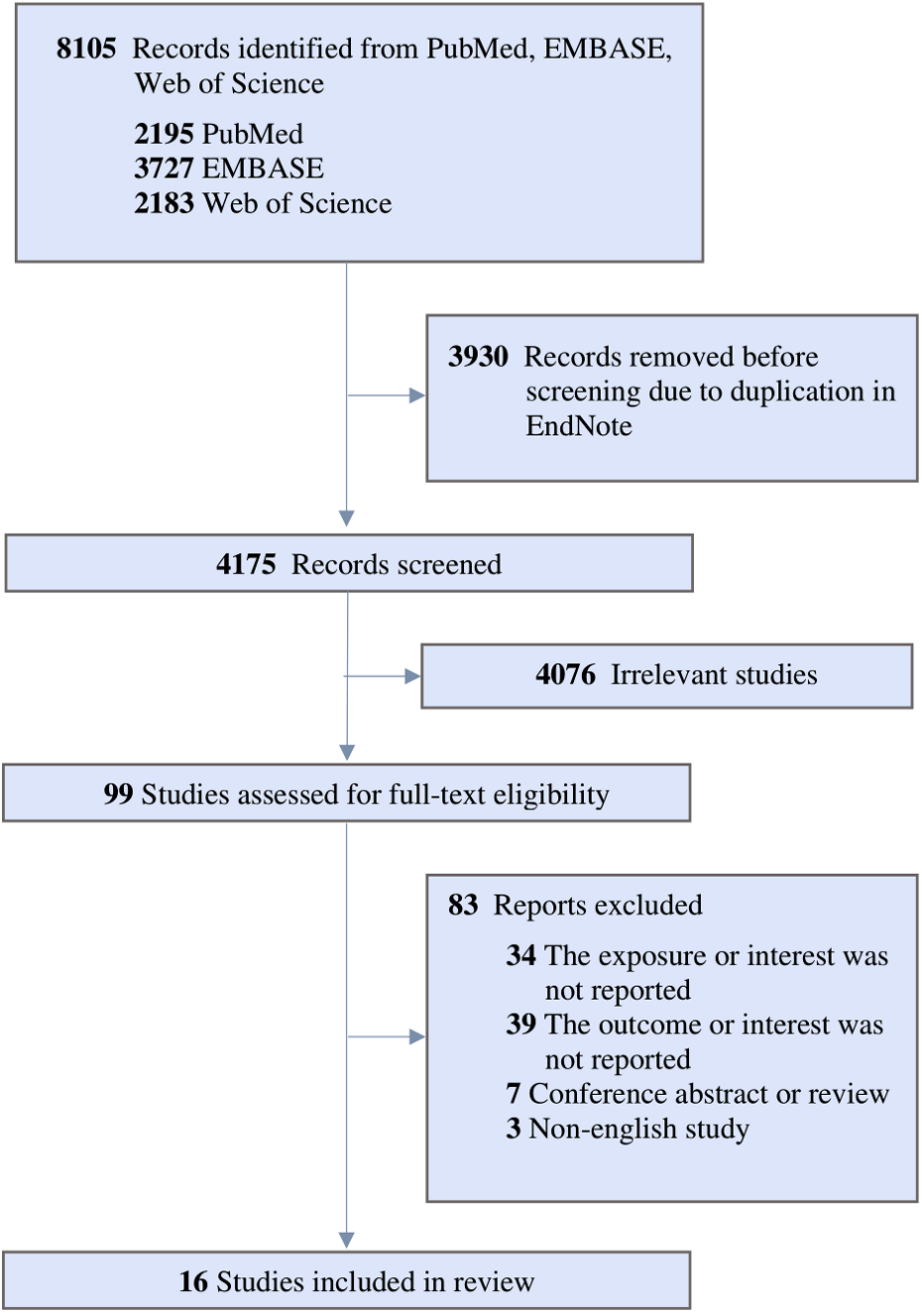
PRISMA diagram of study inclusion.

The collective sample size from the 16 separate datasets consisted of 370,482 pediatric participants. Figure 1 shows the PRISMA diagram depicting the process of study inclusion, which involved a total of 8,105 articles identified through the keyword search strategy. Following the elimination of duplicate articles, we proceeded to screen 4,175 articles based on their titles and abstracts. Out of these, 99 articles qualified for a thorough full-text review, with 83 subsequently excluded based on the predefined exclusion criteria. Ultimately, this meta-analysis encompassed a total of 16 articles in its sample.

All studies were conducted after 2008 with most studies from 2017 through 2022. Characteristics of the included studies are presented in Supplementary Table S3 in the Supplementary Material. The geographic areas covered by the included studies are illustrated in Supplementary Figure S1. In terms of study design, nine (56.25%) articles were time-series studies, two (12.5%) were cross-sectional studies, two (12.5%) were case-crossover studies, two (12.5%) were cohort study, and one (6.25%) was a case-control study. Study locations included countries in Africa, Asia, Europe, Australia, and South America with most being in temperate or tropical climate zones.

### 3.2 Temperature variation increased risk of pediatric pneumonia events

**Figure 2:**
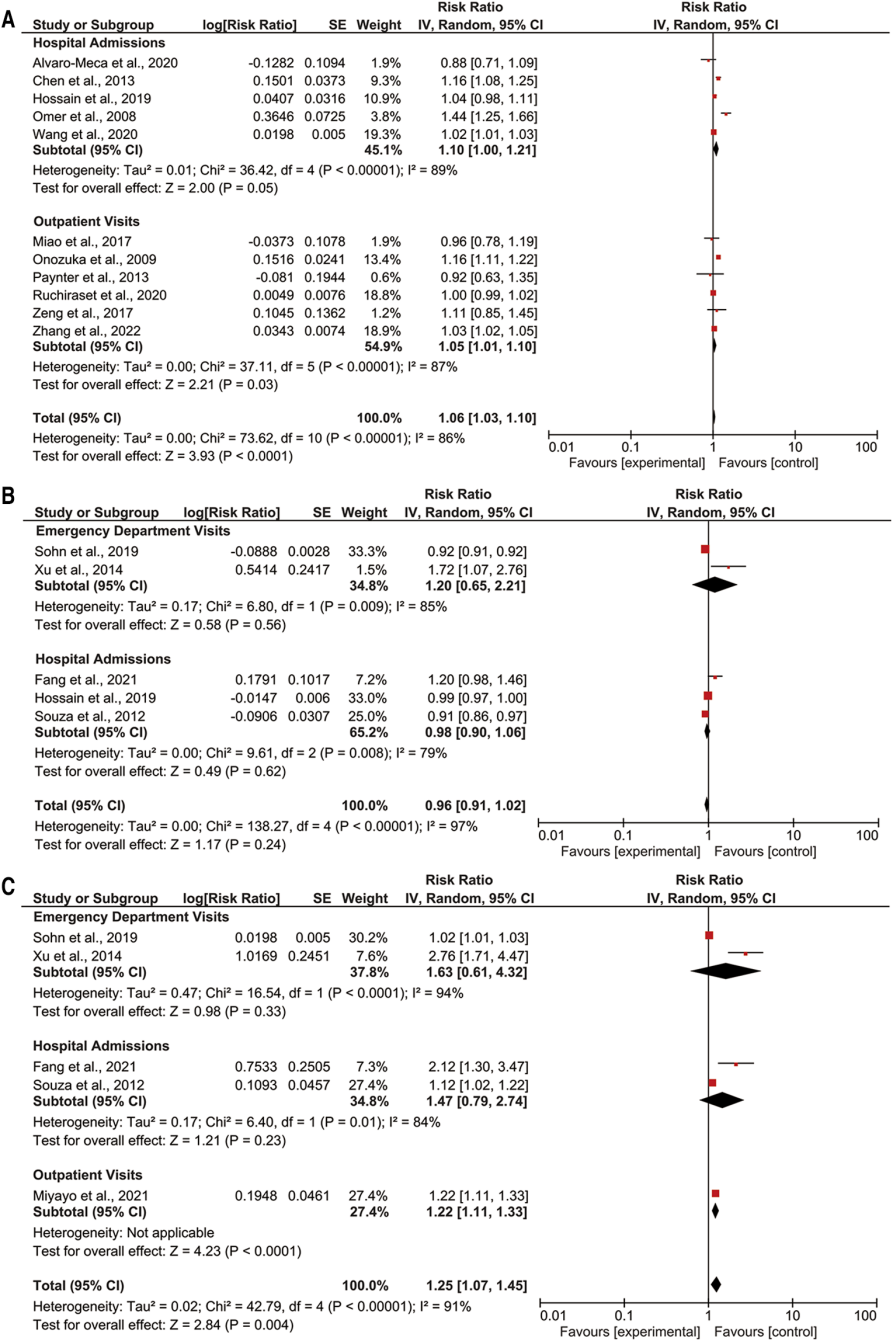
Forest plot of (**A**) temperature variation; (**B**) extreme heat; and (**C**) extreme cold with pediatric pneumonia events.

We observed that a 1°C temperature variation increased the RR by 1.06-fold of the pediatric pneumonia events (95% CI: 1.03–1.10; *p* < 0.05) (Figure 2A). A 1°C temperature variation increased the RR by 1.10-fold of the pediatric pneumonia hospital admission (95% CI: 1.00–1.21; *p* = 0.05). Additionally, a 1°C temperature variation increased the RR by 1.06-fold of the pediatric pneumonia emergency department visits (95% CI: 1.01–1.10; *p* < 0.05). The results for pediatric pneumonia events did not differ after sensitivity analysis (see Supplementary Figure S2 in the Supplementary Material).

### 3.3 Extreme heat not associated with increased risk of pediatric pneumonia events

There was no association observed between extreme heat and the pediatric pneumonia events (Figure 2B). Effect estimates of an increase in extreme heat were not associated with either pediatric pneumonia emergency department visits or hospital admissions. The results for paediatric pneumonia events did not differ after sensitivity analysis (see Supplementary Figure S3 in the Supplementary Material).

### 3.4 Extreme cold was associated with an increased risk of pediatric pneumonia events

We observed an increase in extreme cold increased the RR by 1.25-fold of the pediatric pneumonia events (95% CI: 1.07–1.45; *p* < 0.05) (Figure 2C). Effect estimates of an increase in extreme cold were not associated with either pediatric pneumonia emergency department visits or pediatric pneumonia hospital admissions. An increase in extreme cold was associated with pediatric pneumonia outpatient visits (RR: 1.22; 95% CI: 1.11–1.33; *p* < 0.05). The results for paediatric pneumonia events did not differ after sensitivity analysis (see Supplementary Figure S4 in the Supplementary Material).

### 3.5 Associations of temperature variation with pediatric pneumonia events by age, sex, and climate zone

**Figure 3:**
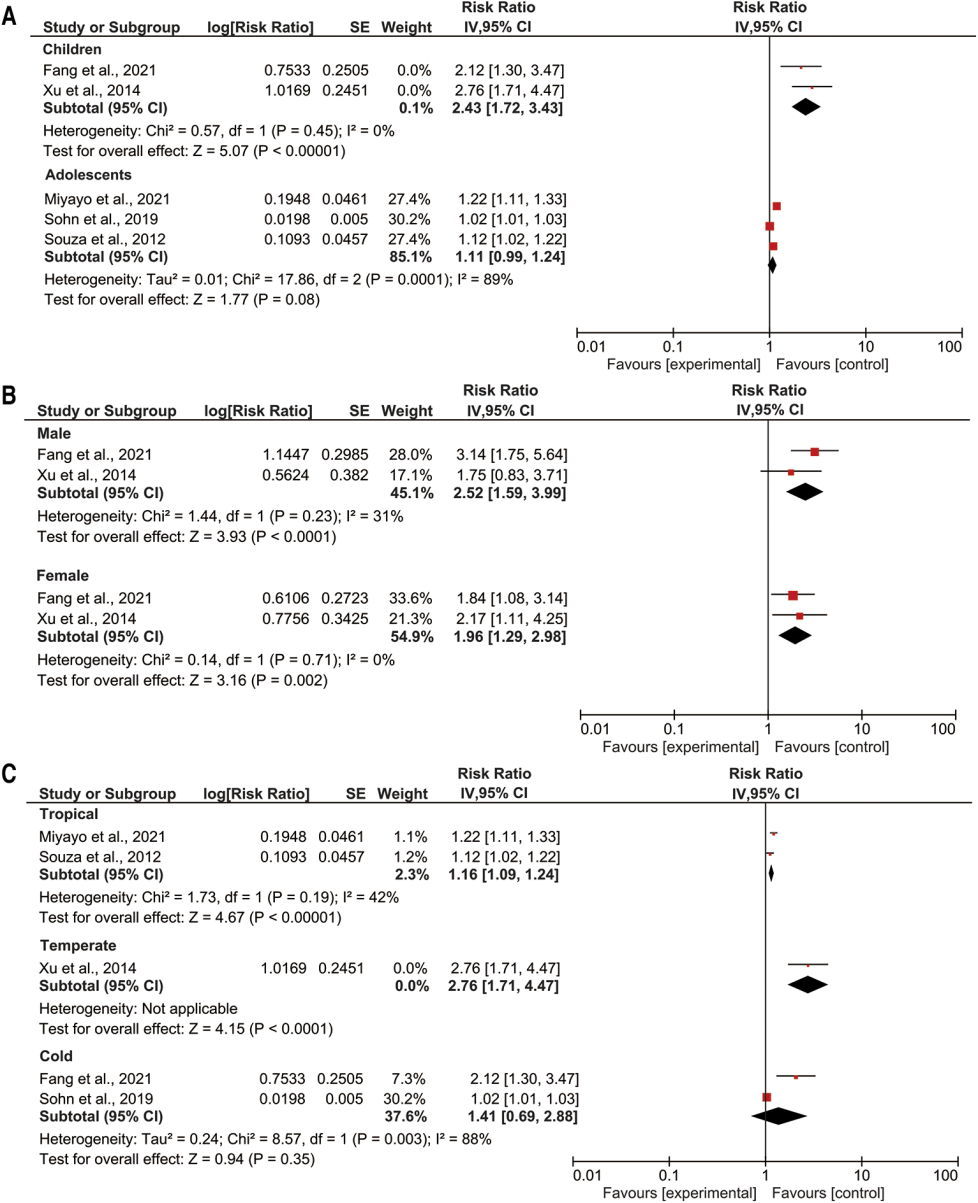
Subgroup analyses of temperature variation with pediatric pneumonia events (**A**) age; (**B**) gender; (**C**) climate zone.

We observed that a 1°C temperature variation increased the RR of pneumonia events in children by 1.19-fold (95% CI: 1.08–1.32; *p* < 0.05) (Figure 3A). Conversely, a 1°C temperature variation and pneumonia events in adolescents were not associated. A 1°C temperature variation increased the RR of pneumonia events by 1.03-fold among girls (95% CI: 1.02–1.05; *p *< 0.05) (Figure 3B). Among boys, a 1°C temperature variation was not associated with pediatric pneumonia events. Additionally, a 1°C temperature variation increased the RR of pneumonia events by 1.07-fold in temperate climate zones (95% CI: 1.03–1.11; *p *< 0.05) (Figure 3C). Temperature variation was not associated with pediatric pneumonia events in arid or tropical climate zones.

### 3.6 Associations of extreme heat with pediatric pneumonia events by age, sex, and climate zone

**Figure 4:**
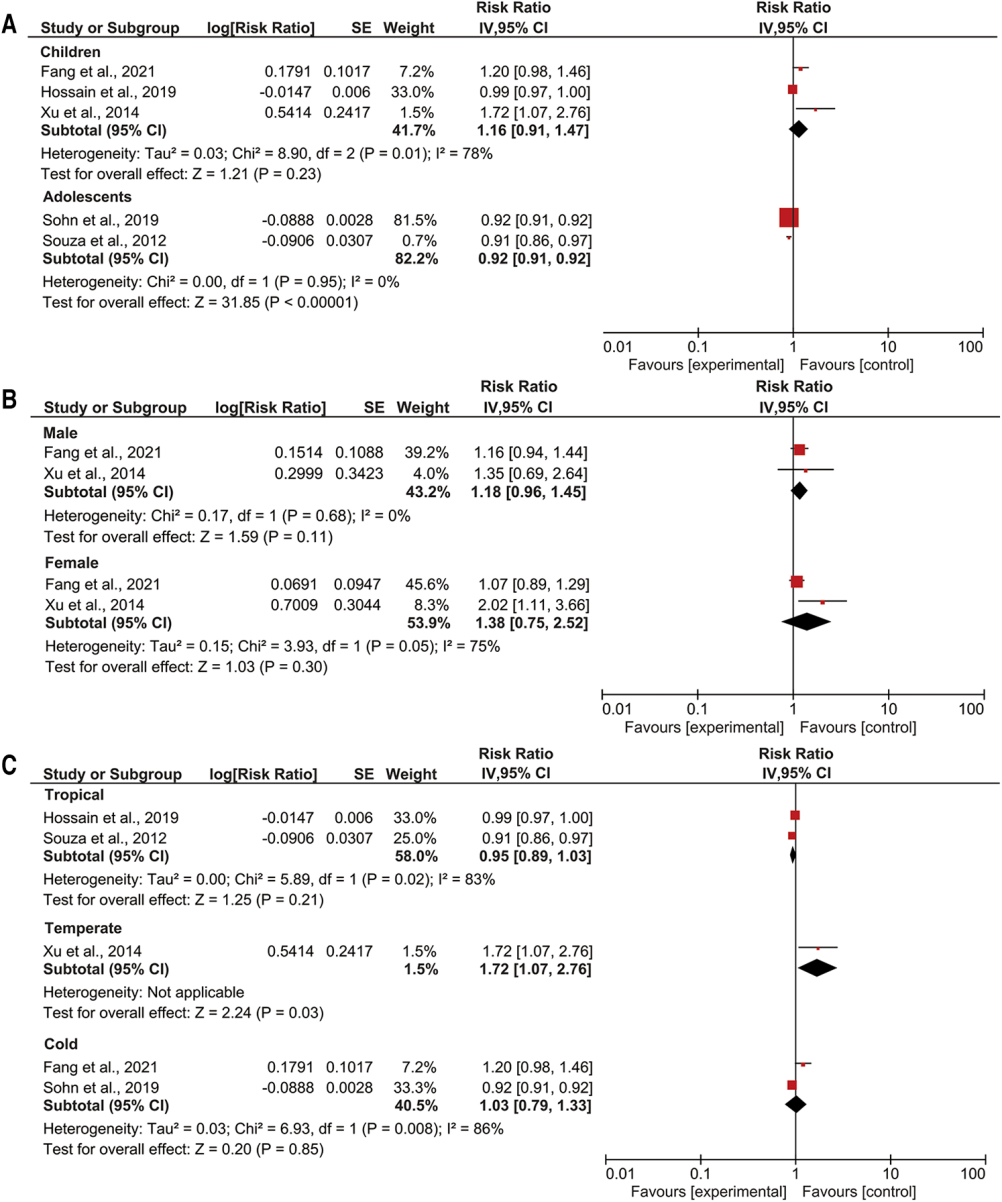
Subgroup analyses of extreme heat with pediatric pneumonia events (**A**) age; (**B**) gender; (**C**) climate zone.

We observed an increase in extreme heat decreased the RR of pneumonia events in adolescents by 0.92-fold (95% CI: 0.91–0.92; *p* < 0.05) (Figure 4A). By contrast, extreme heat and pneumonia events in children were not associated. In subgroup analysis, sex was not associated with pediatric pneumonia events (Figure 4B). In addition, an increase in extreme heat increased the RR of pediatric pneumonia events by 1.72-fold in temperate climate zones (95% CI: 1.07–2.76; *p *< 0.05) (Figure 4C). Extreme heat was not associated with pediatric pneumonia events in tropical or cold climate zones.

### 3.7 Associations of extreme cold with pediatric pneumonia events by age, sex, and climate zone

**Figure 5:**
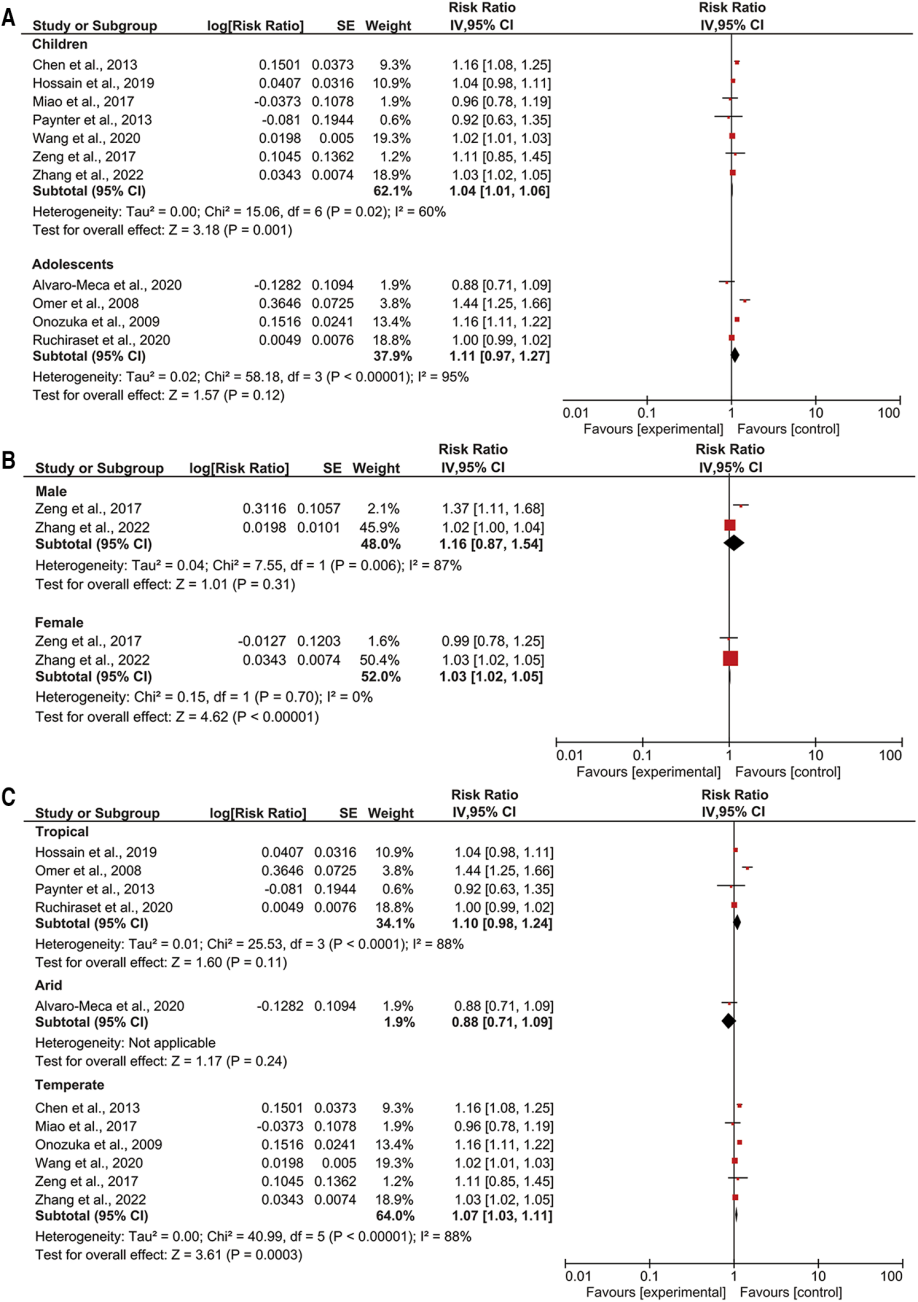
Subgroup analyses of extreme cold with pediatric pneumonia events (**A**) age; (**B**) gender; (**C**) climate zone.

We observed an increase in extreme cold increased the RR of pneumonia events in children by 2.43-fold (95% CI: 1.72–3.43; *p* < 0.05) (Figure 5A). Extreme cold was not associated with pneumonia events in adolescents. An increase in extreme cold increased the RR of pediatric pneumonia events by 2.52-fold among boys (95% CI: 1.59–3.99; *p *< 0.05) and the RR of pediatric pneumonia events by 1.96-fold among girls (95% CI: 1.29–2.98; *p *< 0.05) (Figure 5B). In addition, an increase in extreme cold increased the RR of pediatric pneumonia events by 1.16-fold in tropical climate zones (95% CI: 1.03–1.11; *p *< 0.05) and the RR of pediatric pneumonia events by 2.76-fold in temperate climate zones (95% CI: 1.71–4.47; *p *< 0.05) (Figure 5C). Extreme cold was not associated with paediatric pneumonia events in cold climate zones.

## 4 Discussion

The novelty of this study is that we investigated the effects of variation and extreme of ambient temperature on pediatric pneumonia-related outcomes. Significant findings of this work are that temperature variation and extreme cold increased the risk of pediatric pneumonia events, especially for children (<13 years), for girls, and in temperate climate zones. Our results suggest that extreme weather event could increase the risk of pediatric pneumonia events.

First, we observed that temperature variation increased the risk of pediatric pneumonia events. A report demonstrated that a 1°C increase in temperature variation induced by climate change increased the risk of pediatric pneumonia events by 3.19-fold (95% CI: 1.85–4.54; *p* < 0.05) (22). We also evaluated the effects of temperature variation on pediatric pneumonia hospital admissions and observed that temperature variations are an important risk factor for pediatric pneumonia hospital admissions. A previous study showed that 1°C temperature variation in all seasons for 0–6 days corresponded to a 0.65% increase in pediatric pneumonia hospital admissions (95% CI: 0.34%–0.96%; *p *< 0.05) (37). Consistently, we observed that temperature variation is a critical risk factor for pediatric pneumonia emergency department visits. In Seoul, South Korea, emergency department visits for respiratory tract infections increase by 6.01% (95% CI: 2.45–9.69; *p *< 0.05) among patients aged for 6 to 18 years old for every 1°C change in the diurnal temperature range (38). Taken together, temperature variations contribute to diverse and heightened risks of pediatric pneumonia across various geographical regions.

We observed that extreme cold increased the risk of pediatric pneumonia events. A previous study in Atlanta observed that pneumonia outpatient visits increased by 1.12-fold two weeks after the onset of extreme cold (95% CI: 1.04–1.20; *p* < 0.05) (39). Exposure to cold caused pediatric pneumonia hospital admissions to increase by approximately 5.1% compared with non-cold exposure (*p *< 0.05) (40). Dry air during cold events can lead to the drying and irritation of mucous membranes in the respiratory tract, impairing their protective function and facilitating the entry of viruses and bacteria into the airways (41). We observed that extreme cold is an important risk factor for pediatric pneumonia outpatient visits. One study observed that pneumonia outpatient visits increased by 1.12-fold because of extreme cold (95% CI: 1.04–1.20; *p *< 0.05) (39). Therefore, our findings indicate that extreme cold increases the risk of pediatric pneumonia events.

We did not observe significant associations between extreme cold and the risk of pediatric pneumonia events. High temperatures reduce the transmission efficiency of viruses (42), which may explain our findings. Higher temperatures were not demonstrated to enhance the transmissibility of the pneumonia-causing virus and substantially reduced shedding of the virus (43). One study demonstrated that the RR of pediatric respiratory tract infections decreased by 0.82-fold in extreme heat in Belgium (95% CI: 0.78–0.87; *p* < 0.05) (44). Studies have demonstrated that air transmission efficiency decreases with increased temperature to a level of undetectability at 30°C (45, 46). Therefore, our findings indicate that extreme cold increases the risk of pediatric pneumonia events.

Next, we observed that children are susceptible to pneumonia events caused by temperature variations. One study reported that a 1°C daily temperature variation increased the RR by 1.89-fold of pneumonia events in children aged 0–5 years (95% CI: 1.34–2.67; *p* < 0.05) (47). A possible explanation is that children spend more time outdoors than individuals of other ages are thus exposed more to outdoor temperature (48). Girls had a higher risk of pediatric pneumonia events caused by temperature variations. A study reported that a 1°C temperature variation was associated with a 14%–22% increase in pediatric pneumonia events among girls (49). Female individuals are disproportionately likely to be infected by fungi such as *Mycoplasma*, which are leading causes of community-acquired pneumonia during temperature variations compared to male individuals (50). We observed that individuals in temperate climate zones had a higher risk of pediatric pneumonia events caused by temperature variations. These results are consistent with study undertaken in China that included humid subtropical climate settings (37). One such study observed that pediatric pneumonia events increased by 0.71% with temperature variation (95% CI: 0.38%–1.04%; *p *< 0.05). In addition, total respiratory disease mortality increases from 7.9% to 12.6% in temperate climate zones (51). This could be because of latitude, longitude, or weather that might cause variations in the magnitude of the temperature variation effect on pediatric pneumonia events (37, 52). Taken together, girls, children, and individuals in temperate climate zones are populations-at-risk of pneumonia events caused by temperature variation.

We also observed that extreme heat decreased pneumonia events in adolescents. A Brazilian study obtained the same results and reported that the risk of pneumonia events in adolescents decreased by 0.92-fold during extreme heat (95% CI: 0.91–0.92; *p *< 0.05) (25). A study in Japan showed that *M pneumoniae* pneumonia cases increased with an increase of temperature related to climate change in children (19). Extreme heat events are less frequent and less intense in other regions than in tropical or subtropical regions, where heat is a more persistent and common climate feature (53). Therefore, the populations and healthcare infrastructure in temperate climate zones not be as well adapted to dealing with the health impacts of extreme heat, including the exacerbations of respiratory conditions such as pneumonia (54). Further, it appears that the metabolism of children does not adapt as effectively as that of adults to heat stress (55). Consequently, extreme heat may affect pneumonia events in adolescents and individuals in temperate climate zones.

Children are the most vulnerable group for pneumonia events caused by extreme cold. A study reported that extreme cold increased the risk of pneumonia events in children by 1.06-fold (95% CI: 0.98–1.14; *p *< 0.05) (56). Another study reported that children will be more vulnerable to sharp temperature decreases in the future than in the past if unstable weather patterns occur because of climate change (10). Girls were a population at risk of pediatric pneumonia events identified in our study. A study showed that extreme cold increased the risk of pneumonia events in female individuals by 1.84-fold (95% CI 1.08–3.14; *p *< 0.05) (24). Cold temperatures increase the production of stress hormones, especially in women and girls, which suppress the immune system and make it more difficult for the body to fight infections (57). We identified that extreme cold increased the risk of pediatric pneumonia events in individuals in temperate and tropical climate zones. These results are consistent with those of studies in Brazil and China, which reported that pediatric pneumonia events increased during extreme cold events compared with during non-extreme cold events (58, 59). Children who live in temperate and tropical climates zones are not accustomed to extreme cold, and their bodies may experience shock on exposure (2, 8). This can compromise their immune system, making them more susceptible to pneumonia. Therefore, extreme cold could contribute to an increased risk of pneumonia events.

This study has some limitations. Firstly, the included studies lacked detailed data, precluding the display of all analyses for specific subgroups. Secondly, the temperature measurements in these studies were derived from either centrally located monitoring station data or personal exposure assessments. This methodological variability may have introduced geographical biases and influenced estimations of individual exposure. Consequently, the identification of specific effect modifiers may be limited, considering potential geographical variability and biases inherent in exposure assessment methods.

## 5 Conclusion

In conclusion, exposure to variation and extreme ambient temperature was associated with pediatric pneumonia-related events. Pneumonia appears to be more influenced by temperature-mediated climate change among children, particularly girls, and individuals residing in temperate climate zones. Extreme temperature events mediated by climate change should be prioritized in future environmental policies and pediatric pneumonia prevention.

## Data Availability

The original contributions presented in the study are included in the article/Supplementary Material, further inquiries can be directed to the corresponding author/s.
