# Supplementary material for: Extreme temperatures increase the risk of pediatric pneumonia: a systematic review and meta-analysis
Source: Front Pediatr. 2024 Feb 2;12:1329918. doi: 10.3389/fped.2024.1329918 (PMC10869493; doi:10.3389/fped.2024.1329918)
Supplement: Supplementary file 1 [file Datasheet1.docx]

**SUPPLEMENTARY INFORMATION**

**Extreme temperatures increase the risk of pediatric pneumonia: A systematic review and meta-analysis**

Firdian Makrufardi^1,2^, Rina Triasih^2^, Nurnaningsih^2^, Kian Fan Chung^3^, Sheng-Chieh Lin^4,5*^, Hsiao-Chi Chuang^3,6,7,8,9*^

^1^International Ph.D. Program in Medicine, College of Medicine, Taipei Medical University, Taipei, Taiwan

^2^Department of Child Health, Faculty of Medicine, Public Health, and Nursing, Universitas Gadjah Mada – Dr. Sardjito Hospital, Yogyakarta, Indonesia

^3^National Heart and Lung Institute, Imperial College London, London, United Kingdom

^4^Department of Pediatrics, School of Medicine, College of Medicine, Taipei Medical University, Taipei, Taiwan

^5^Division of Allergy, Immunology, and Rheumatology, Department of Pediatrics, Shuang Ho Hospital, Taipei Medical University, New Taipei City, Taiwan

^6^School of Respiratory Therapy, College of Medicine, Taipei Medical University, Taipei, Taiwan

^7^Division of Pulmonary Medicine, Department of Internal Medicine, Shuang Ho Hospital, Taipei Medical University, New Taipei City, Taiwan

^8^Cell Physiology and Molecular Image Research Center, Wan Fang Hospital, Taipei Medical University, Taipei, Taiwan

^9^Graduate Institute of Medical Sciences, College of Medicine, Taipei Medical University, Taipei, Taiwan

**Corresponding authors:**Sheng-Chieh Lin, MD, PhD

Department of Pediatrics, School of Medicine, College of Medicine, Taipei Medical University, 250 Wuxing Street, Taipei 11031, Taiwan.

Telephone: +886-2-22490088 ext. 2951. Fax: +886-2-22490088. E-mail: [jacklinbox](mailto:r92841005@ntu.edu.tw)@tmu.edu.tw

Hsiao-Chi Chuang, PhD

Inhalation Toxicology Research Lab (ITRL), School of Respiratory Therapy, College of Medicine, Taipei Medical University, 250 Wuxing Street, Taipei 11031, Taiwan.

Telephone: +886-2-27361661 ext. 3513. Fax: +886-2-27391143. E-mail: [chuanghc@tmu.edu.tw](mailto:r92841005@ntu.edu.tw)

**List of Tables**

Table S1 Search strategies used for online databases 4

Table S2. Quality assessment and risk of bias of included studies for meta-analysis 5

Table S3. Characteristics of studies that included in systematic review and meta-analysis 7

**List of Figures**

Figure S1. Regions covered by the studies included 3

Figure S2. Forest plot with sensitivity analysis for temperature variation with pediatric pneumonia events. 11

Figure S3. Forest plot with sensitivity analysis for extreme heat with pediatric pneumonia events. 12

Figure S4. Forest plot with sensitivity analysis for extreme cold with pediatric pneumonia events. 13


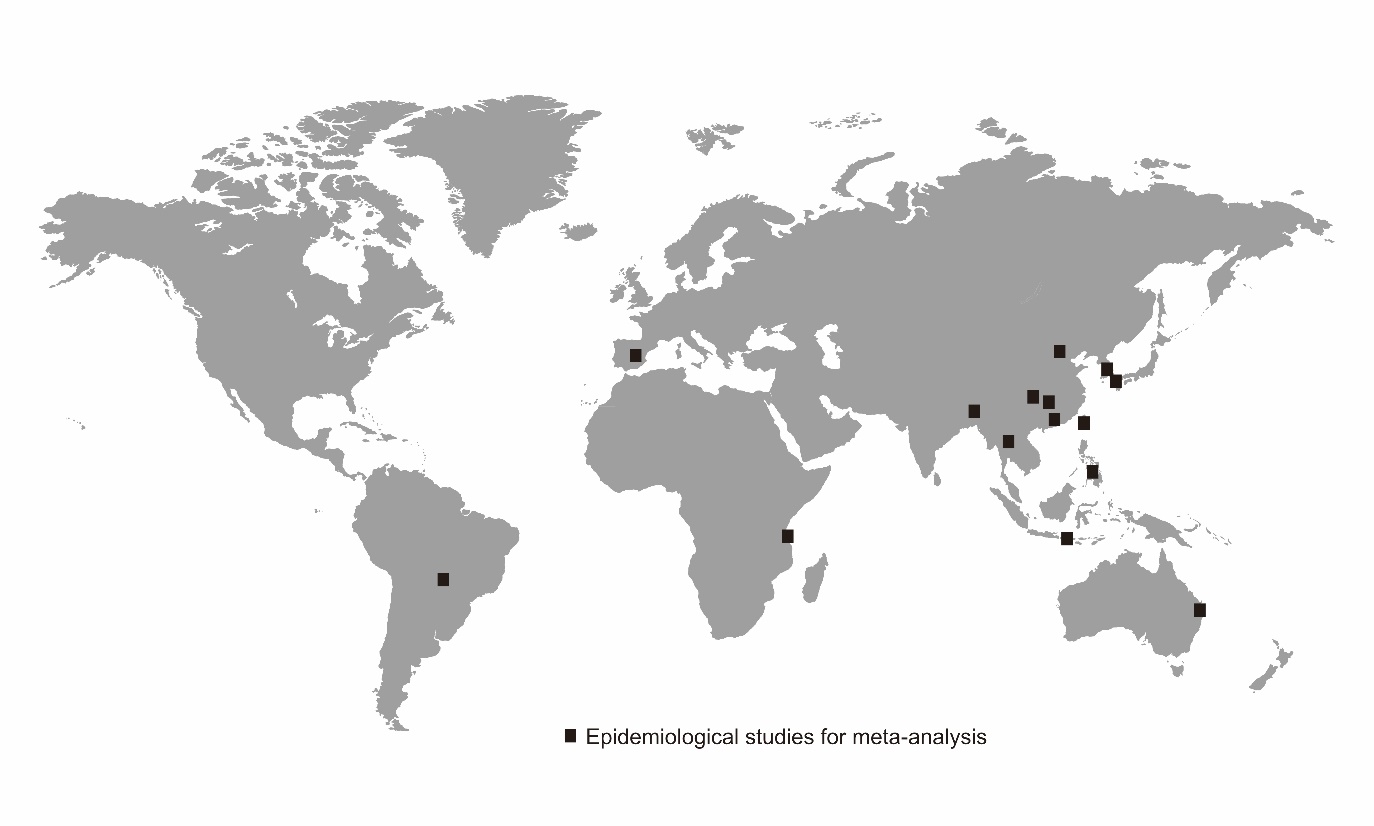


**Figure S1.** Regions covered by the studies included.

**Table S1. Search strategies used for online databases**

| **Database** | **Search strategies** |
| --- | --- |
| **PubMed** | (“pneumonia”[Title/Abstract] OR “respiratory tract infection”[Title/Abstract]) AND (“meteorology”[Title/Abstract] OR “temperature”[Title/Abstract] OR “extreme temperature”[Title/Abstract] OR “extreme heat”[Title/Abstract] OR “extreme cold”[Title/Abstract] OR “temperature variation”[Title/Abstract] OR “ambient temperature”[Title/Abstract] OR “inter-day temperature variability”[Title/Abstract] OR “diurnal temperature range”[Title/Abstract] OR “temperature changes between neighbouring days”[Title/Abstract] OR “temperature variation between neighbouring days”[Title/Abstract]) |
| **Web of Science** | (TS=(pneumonia OR respiratory tract infection)) AND (TS=(meteorology OR temperature OR extreme temperature OR extreme heat OR extreme cold OR temperature variation OR ambient temperature OR inter-day temperature variability OR diurnal temperature range OR temperature changes between neighbouring days OR temperature variation between neighbouring days)) |
| **Medline and EMBASE** | 1) (pneumonia or respiratory tract infection).mp. [mp=title, abstract, original title, name of substance word, subject heading word, floating sub-heading word, keyword heading word, protocol supplementary concept word, rare disease supplementary concept word, unique identifier, synonyms]  2) (meteorology or temperature or extreme temperature or extreme heat or extreme cold or temperature variation or ambient temperature or inter-day temperature variability or diurnal temperature range or temperature changes between neighbouring days or temperature variation between neighbouring days).mp. [mp=title, abstract, original title, name of substance word, subject heading word, floating sub-heading word, keyword heading word, protocol supplementary concept word, rare disease supplementary concept word, unique identifier, synonyms]  3) 1 and 2 |

# Table S2. Quality assessment and risk of bias of included studies for meta-analysis

| **ID** | **Study (Publication year)** |  | | | | | | | |  |
| --- | --- | --- | --- | --- | --- | --- | --- | --- | --- | --- |
|  |  | **Were the criteria for inclusion in the sample clearly defined?** | **Were the study subjects and the setting described in detail?** | **Was the exposure measured in a valid and reliable way?** | **Were objective, standard criteria used for measurement of the condition?** | **Were confounding factors identified?** | **Were strategies to deal with confounding factors stated?** | **Were the outcomes measured in a valid and reliable way?** | **Was appropriate statistical analysis used?** | **Total** |
|  | Alvaro-Meca, et al. (2020) | Yes | Yes | Yes | Yes | Yes | No | Yes | Yes | 7 |
|  | Chen, et al. (2013) | Yes | Yes | Yes | Yes | Yes | No | Yes | Yes | 7 |
|  | Fang, et al. (2021) | Yes | Yes | Yes | Yes | Yes | No | Yes | Yes | 7 |
|  | Hossain, et al. (2019) | Yes | Yes | Yes | Yes | Yes | Yes | Yes | Yes | 8 |
|  | Miao, et al. (2017) | Yes | Yes | Yes | Yes | Yes | Yes | Yes | Yes | 8 |
|  | Miyayo, et al. (2019) | Yes | Yes | Yes | Yes | No | No | Yes | Yes | 6 |
|  | Omer, et al. (2008) | Yes | Yes | Yes | Yes | Yes | No | Yes | Yes | 7 |
|  | Onozuka, et al. (2009) | Yes | Yes | Yes | Yes | Yes | No | Yes | Yes | 7 |
|  | Paynter, et al. (2013) | Yes | Yes | Yes | Yes | Yes | No | Yes | Yes | 7 |
|  | Ruchiraset, et al. (2020) | Yes | Yes | Yes | Yes | Yes | No | Yes | Yes | 7 |
|  | Sohn, et al. (2019) | Yes | Yes | Yes | Yes | Yes | Yes | Yes | Yes | 8 |
|  | Souza, et al. (2012) | Yes | Yes | Yes | Yes | Yes | No | Yes | Yes | 7 |
|  | Wang, et al. (2020) | Yes | Yes | Yes | Yes | Yes | Yes | Yes | Yes | 8 |
|  | Xu, et al (2014) | Yes | Yes | Yes | Yes | Yes | Yes | Yes | Yes | 8 |
|  | Zeng, et al. (2017) | Yes | Yes | Yes | Yes | Yes | Yes | Yes | Yes | 8 |
|  | Zhang, et al. (2022) | Yes | Yes | Yes | Yes | Yes | Yes | Yes | Yes | 8 |

Note: * Risk of bias assessment was accessed by the Joanna Briggs Institute (JBI);

*Moola S, Munn Z, Tufanaru C, Aromataris E, Sears K, Sfetcu R, Currie M, Qureshi R, Mattis P, Lisy K, Mu P-F. Chapter 7: Systematic reviews of etiology and risk . In: Aromataris E, Munn Z (Editors). JBI Manual for Evidence Synthesis. JBI, 2020. Available from https://synthesismanual.jbi.global*

# Table S3. Characteristics of studies that included in systematic review and meta-analysis

| **ID** | **Reference**  **(year)** | **Location (continent)** | **Study period** | **Study design** | **Subgroup age** | **Sample size** | **Outcome** | **Effect estimates**  **(95%CI)** | **Subgroup analysis** |
| --- | --- | --- | --- | --- | --- | --- | --- | --- | --- |
|  | Alvaro-Meca, et al (2020) | Spain | January-December 2013 | Case-crossover | Adolescents | 67443 | Pneumonia  ICD-9-CM codes: 480.x, 481.x, 482.x, 483.x, 484.x, 485.x, 486.x, 487.0 | **Temperature variation**  OR = 0.88 (95% CI: 0.71-1.09) |  |
|  | Chen et al (2013) | Taipei, Taiwan | 2006-2010 | Case-crossover | Children | 8157 | Pneumonia  ICD-  9, (480–487) | **Temperature variation**  RR = 1.16 (95% CI: 1.08-1.25) |  |
|  | Fang et al (2021) | Beijing, China | 2013-2017 | Time series | Children | 22169 | Pneumonia  ICD 10-AM codes (J12–J18, J69) | **Extreme cold**  RRs = 2.12 (95% CI: 1.30, 3.47)  **Extreme heat**  RRs = 1.20 (95% CI: 0.98, 1.46) | **Extreme heat**  **Gender:**  Female: 1.07 (95% CI: 0.89-1.29)  Male: 1.16 (95% CI: 0.94-1.44)  **Extreme cold**  **Gender:**  Female: 1.84 (95% CI: 1.08-3.14)  Male: 3.14 (95% CI: 1.75-5.64) |
|  | Hossain et al (2019) | Matlab, Bangladesh | 2012-2016 | Time series | Children | 2743 | Pneumonia  ICD10-AM codes J10.0–J18.9 | **Extreme heat**  RR **=** 0.99 (95% CI: 0.97–1.00)  **Temperature variation**  RR = 1.04 (95% CI: 0.98-1.11) |  |
|  | Miao et al (2017) | Changsa, China | 2011-2012 | Cross-sectional | Children | 2598 | Pneumonia  ICD10-AM codes J10.0–J18.9 | **Temperature variation**  OR = 0.96 (95% CI: 0.78-1.19) |  |
|  | Miyayo et al (2019) | Tanga, Tanzania | 2016-2018 | Time series | Adolescents | 8579 | Pneumonia | **Extreme cold**  RR = 1.21 (95% CI: 1.11-1.33) |  |
|  | Omer et al (2008) | Lombok, Indonesia | 2000-2002 | Cross-sectional | Adolescents | 2878 | Pneumonia  ICD-  9, (480–487) | **Temperature variation**  RR = 1.44 (95% CI: 1.24–1.66) |  |
|  | Onozuka et al (2009) | Fukuoka, Japan | 1999-2007 | Time series | Adolescents | 13056 | Pneumonia | **Temperature variation**  RR = 1.16 (95% CI: 1.11-1.22) |  |
|  | Paynter et al (2013) | Tagbilaran, Philippines | 2000-2004 | Time series | Children | 2187 | Pneumonia | **Temperature variation**  RR = 0.93 (95% CI: 0.63–1.35) |  |
|  | Ruchiraset et al (2020) | Lampang Province, Thailand | 2003-2014 | Time series | Adolescents | 41085 | Pneumonia (ICD-10; J12, J13, J14, J15, J16, and J18). | **Temperature variation**  RR = 1.00 (95% CI: 0.99–1.02) |  |
|  | Sohn et al (2019) | Seoul, Republic of Korea | 2009-2014 | Time series | Adolescents | 113243 | Pneumonia (ICD-10; J12-J18) | **Extreme cold**  RR = 1.02 (95% CI: 1.01-1.03)  **Extreme heat**  RR = 0.92 (95% CI: 0.91-0.92) |  |
|  | Souza et al (2012) | Campo Grande, Brazil | 2004-2008 | Time series | Adolescents | 6630 children, 2866 infants | Pneumonia  ICD-  9, (480–487 | **Extreme cold**  ETmin: RR 1.12 (95% CI: 1.02-1.22)  **Extreme heat**  ETmax: RR 0.91 (95% CI: 0.86-0.97) |  |
|  | Wang et al (2020) | Chongqing, China | 2009-2018 | Prospective cohort | Children | 6611 | Pneumonia | **Temperature variation**  OR = 1.02 (95% CI: 1.01‒1.03) |  |
|  | Xu et al (2014) | Brisbane, Australia | 2001-2010 | Case control study | Children | 17238 | Pneumonia  (ICD-9 codes: 480–486; ICD-10 codes: J12–J18) | **Extreme heat**  Lag 0-1: 1.07 (95% CI: 0.92-1.26), Lag 0-13: 1.33 (95% CI: 0.93-1.91), Lag **0-21**: Lag 0-21: 1.72 (95% CI: 1.07-2.76)  **Extreme cold**  Lag 0-1: 0.89 (95% CI: 0.77,1.03), Lag 0-13: 1.30 (95% CI: 0.91,1.86), Lag 0-21: 2.76 (95% CI: 1.71,4.47) | **Extreme heat:**  **(0,1):** Lag 0-1: 0.95(95% CI: 0.62-1.44), Lag 0-13: 1.02 (95% CI: 0.42-2.49), Lag 0-21: 0.99 (95% CI: 0.31-3.18)  **(1,2):** Lag 0-1: 0.94 (95% CI: 0.69-1.29), Lag 0-13: 1.27 (95% CI: 0.74-2.18), Lag 0-21: 2.04 (95% CI: 1.01-4.11)  **(5,14):** Lag 0-1: 1.18 (95% CI: 0.89-1.57), Lag 0-13: 1.42 (95% CI: 0.72-2.78), Lag 0-21: 1.14 (95% CI: 0.46-2.81)  **Male:** Lag 0-1: 0.96 (95% CI: 0.76-1.21), Lag 0-13: 1.11 (95% CI: 0.66-1.86), Lag 0-21: 1.35 (95% CI: 0.69-2.64)  **Female:** Lag 0-1: 1.17 (95% CI: 0.96-1.42), Lag 0-13: 1.50 (95% CI: 0.95-2.36), Lag 0-21: 2.02 (95% CI: 1.11-3.66)  **Extreme cold**  **(0,1):** Lag 0-1: 1.10 (95% CI: 0.77,1.56), Lag 0-13: 1.08 (95% CI: 0.43,2.69), Lag 0-21: 1.87 (95% CI: 0.55,6.32)  **(1,2):** Lag 0-1: 0.92(95% CI: 0.71,1.21), Lag 0-13: 1.33 (95% CI: 0.72,2.81), Lag 0-21: 1.91 (95% CI: 0.76,4.76)  **(5,14):** Lag 0-1: 0.78 (95% CI: 0.63-1.01), Lag 0-13: 1.37 (95% CI: 0.80-2.36), Lag 0-21: 3.03 (95% CI: 1.47-6.26)  **Male:** Lag 0-1: 1.00 (95% CI: 0.76-1.31), Lag 0-13: 1.46 (95% CI: 0.73-2.88), Lag 0-21: 1.75 (95% CI: 0.83-3.71)  **Female:** Lag 0-1: 0.88 (95% CI: 0.72-1.07), Lag 0-13: 1.11 (95% CI: 0.67-1.82), Lag 0-21: 2.17 (95% CI: 1.11-4.25) |
|  | Zeng et al (2017) | Changsha, China | 2011-2012 | Prospecitve cohort | Children | 2598 | Pneumonia | **Temperature variation**  OR = 1.11 (95% CI: 0.85-1.45) | **Male:** 1.36 (95% CI: 1.11, 1.66)  **Female:** 0.99 (95% CI: 0.78, 1.25) |
|  | Zhang et al (2022) | Guangzhou, China | 2013-2019 | Time series | Children | 50401 | Pneumonia (ICD-10; J12-J18) | **Temperature variation**  RR = 1.03 (95% CI: 1.02-1.05)  **Female**  RR = 1.03 (95% CI: 1.02-1.05)  **Male**  RR = 1.02 (95% CI: 1.00-1.04) |  |


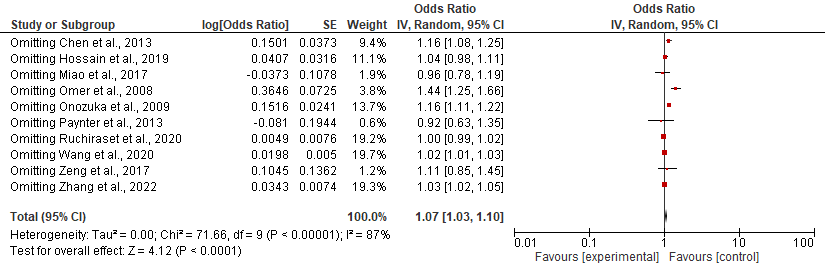


**Figure S2.** Forest plot with sensitivity analysis for temperature variation with pediatric pneumonia events.


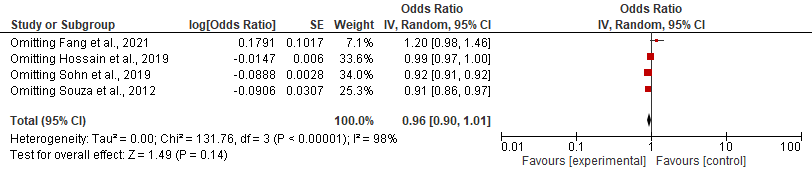


**Figure S3.** Forest plot with sensitivity analysis for extreme heat with pediatric pneumonia events.


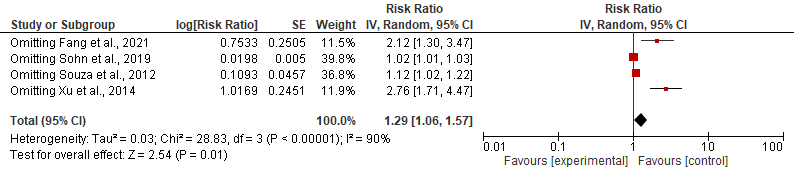


**Figure S4.** Forest plot with sensitivity analysis for extreme cold with pediatric pneumonia events.
